# Increase in HIV-1-transmitted drug resistance among ART-naïve youths at the China-Myanmar border during 2009 ~ 2017

**DOI:** 10.1186/s12879-021-05794-5

**Published:** 2021-01-21

**Authors:** Yibo Ding, Min Chen, Jibao Wang, Yuecheng Yang, Yi Feng, Lijie Wang, Song Duan, Qianru Lin, Hui Xing, Yanling Ma, Mengjie Han, Liying Ma

**Affiliations:** 1 State Key Laboratory of Infectious Disease Prevention and Control, National Center for AIDS/STD Control and Prevention, Collaborative Innovation Center for Diagnosis and Treatment of Infectious Diseases, Chinese Center for Disease Control and Prevention, 155 Changbai Road, Changping District, Beijing, 102206 China; 2 Institute for AIDS/STD Control and Prevention, Yunnan Center for Disease Control and Prevention, No. 158, Dongsi Street, Xishan District, Kunming, 650022 Yunnan Province China; 3 Dehong Dai and Jingpo Autonomous Prefecture Center for Disease Control and Prevention, Mangshi, 678400 China

**Keywords:** HIV, Transmitted drug resistance, CD4 count, Hotspots

## Abstract

**Background:**

HIV-transmitted drug resistance (TDR) is found in antiretroviral therapy (ART)-naïve populations infected with HIV-1 with TDR mutations and is important for guiding future first- and second-line ART regimens. We investigated TDR and its effect on CD4 count in ART-naïve youths from the China-Myanmar border near the Golden Triangle to better understand TDR and effectively guide ART.

**Methods:**

From 2009 to 2017, 10,832 HIV-1 infected individuals were newly reported along the Dehong border of China, 573 ART-naïve youths (16 ~ 25 y) were enrolled. CD4 counts were obtained from whole blood samples. HIV *pol* gene sequences were amplified from RNA extracted from plasma. The Stanford REGA program and jpHMM recombination prediction tool were used to determine genotypes. TDR mutations (TDRMs) were analyzed using the Stanford Calibrated Population Resistance tool.

**Results:**

The most common infection route was heterosexuals (70.51%), followed by people who inject drugs (PWID, 19.20%) and men who have sex with men (MSM) (8.90%). The distribution of HIV genotypes mainly included the unique recombinant form (URF) (44.08%), 38.68% were CRFs, 13.24% were subtype C and 4.04% were subtype B. The prevalence of TDR increased significantly from 2009 to 2017 (3.48 to 9.48%) in ART-naïve youths (4.00 to 13.16% in Burmese subjects, 3.33 to 5.93% in Chinese subjects), and the resistance to non-nucleoside reverse transcriptase inhibitors (NNRTIs), nucleoside and nucleotide reverse transcriptase inhibitors (NRTIs), and protease inhibitors (PIs) were 3.49, 2.62, and 0.52%, respectively. Most (94.40%, *n* = 34) of HIV-1-infected patients with TDRM had mutation that conferred resistance to a single drug class. The most common mutations Y181I/C and K103N, were found in 7 and 9 youths, respectively. The mean CD4 count was significantly lower among individuals with TDRMs (373/mm^3^ vs. 496/mm^3^, *p* = 0.013).

**Conclusions:**

The increase in the prevalence of HIV-1 TDR increase and a low CD4 count of patients with TDRMs in the China-Myanmar border suggests the need for considering drug resistance before initiating ART in HIV recombination hotspots.

**Supplementary Information:**

The online version contains supplementary material available at 10.1186/s12879-021-05794-5.

## Background

A total of 37.9 million people living with HIV (PLWH) worldwide, and 24.5 million people were receiving antiretroviral therapy (ART) at the end of 2018 [1]. However, the problem of drug resistance (DR) has been a focus with the increasing use of ART. DR, including transmitted drug resistance (TDR) and acquired drug resistance (ADR), develops because of viral replication in the presence of ART drugs. TDR is found in ART-naïve populations infected with the virus carrying DR mutations. TDR surveys effectively guide future first- and second-line ART regimens [2].

The monitoring of population levels of DR is also critical to achieving the WHO/UNAIDS 90–90–90 targets. The WHO HIV drug resistance 2019 report noted that 12 of the 18 countries that reported survey findings of TDR had reached levels above 10%. The 2019 report also reported that the average TDR of the three countries bordering China exceeded the 5% moderate level: Myanmar 5.40% (3.10% ~ 9.20%), Vietnam 5.80% (3.40% ~ 9.50%), and Nepal 12.90% (8.80% ~ 18.50%). Overall, the average TDR among newly reported HIV individuals is relatively low (4.10%) in most recently research in China [3]. Another national survey in 2015 reported that [4], the average TDR of Chinese youth was 3.6% (32/894), including 3.00% of MSM and 5.80% of heterosexual transmission. However, estimates of the rates of TDR in the HIV epidemic vary throughout China. Several surveys reported that the TDR of some cities in Yunnan Province of China exceeded the WHO 5% moderate prevalence level [5, 6].

Yunnan Province is in the southwestern part of China, bordering Myanmar, Vietnam, and Laos. By the end of 2016, the number of PLWH in Yunnan (91,986) was the second-highest of all provinces in China; of these, 70,577 (76.7%) were receiving ART. Dehong city is a major city for trading in the Yunnan-Myanmar border area. Dehong shares an international border with Kachin and the Shan state, Myanmar, which are two of the major states of the “Golden Triangle”. The “Golden Triangle” is one of the world’s largest drug production centers [7], and there is a severe HIV transmission problem around this area. The first HIV spread in Chinese people who inject drugs (PWID) was found in Dehong [8].

The prevalent HIV-1 stains (92.3%) in most regions of China are CRF01_AE, CRF07_BC, subtype B′/B, CRF08_BC. Subtype C, URFs or other CRFs were less than 3% [9]. However, Dehong City is a hotspot of HIV recombination and transmission, [10] from which most of the HIV-1 strains currently circulating in China first appeared [11–14]. In a previous survey of HIV-1-infected youths in Yunnan found that the URF were as high as 64% [15]. Whether frequent recombination increases the spread of drug resistance in treatment-naïve population is not clear. It is essential to monitor TDR in this recombination and transmitted hotspots.

According to the WHO HIV drug resistance (HIVDR) threshold survey method [16], untreated youths (< 25 y) are more likely to have recent infection and are representative of TDR [17, 18]. To better understand TDR and its effect on CD4 count, we investigated TDRMs and HIV genotypes in youths over a 9-year period and examined the effect of TDRMs on CD4 counts in the China-Myanmar border near the “Golden Triangle”.

## Methods

### Study population and ethical review

The ethical review of this study was formally approved by and obtained written consent (approval no. X190111549) from the Medical Ethics Certification Committee of the Chinese Center for Disease Control and Prevention. Written informed consent was obtained from all subjects included in the study.

From 2009 to 2017, a total of 10,832 people were newly reported with HIV-1 infection at the Dehong border of China. Among these individuals, 2210 were youths (< 25 y). We amplified the HIV-1 *pol* gene from the plasma of 666 of these youths who were chosen according to the following criteria: 1) 16 ~ 25 y, newly diagnosed with HIV within 3–6 months, and not mother-to-infant transmission; 2) never received ART; 3) agreed to provide written informed consent and to allow plasma samples to be collected and stored for follow-up studies; and 4) agreed to provide epidemiological information. Finally, 573 (86%, 573/666) youths from whom the viral *pol* gene was successfully amplified were used in the analysis. The annual sampling percentage for this study, were 13.58% in 2009 ~ 2011, 22.65% in 2012 ~ 2013, 26.90% in 2014 ~ 2015, and 52.25% in 2016 ~ 2017 (supplemental Table S1).

### CD4 count and *pol* gene amplification

CD4+ T lymphocyte were counted using FACSCalibur flow cytometer and supporting kits (BD Bioscience, United States). The CD4 counts of 461 (80%) of the youths in the study were recorded. RNA was extracted from 140 μL of plasma samples using the QIAamp RNA Blood Mini Kit (Qiagen, Hilden, Germany). The partial *pol* gene, including the protease and reverse transcriptase (PR and RT) coding regions, was amplified by nested polymerase chain reaction (PCR) and an “in-house” method [19] by one-step RT-PCR, using the primers F1a (TGAARGAITGYACTGARAGRCAGGCTAAT), F1b (ACTGARAGRCAGGCTAATTTTTTAG), and RT-R1 (ATCCCTGCATAAATCTGACTTGC). The primers used in nested PCR were PRT-F2 (CTTTARCTTCCCTCARATCACTCT) and RT-R2 (CTTCTGTATGTCATTGACAGTCC). PR/RT covered a fragment of 1056 bps corresponding to nucleotides 2259 to 3314 relative to the HXB2 genome. The positive PCR amplicons were purified using the QIAquick Gel Extraction Kit (Qiagen, Valencia, CA) and sequenced. Amplicons were purified using the Illustra GFXR PCR DNA and Gel Band Purification Kit (GE Healthcare, United Kingdom) according to the manufacturer’s recommendations. The purified DNA was sequenced using the Big Dye Terminator Cycle Sequencing Ready Reaction Kit v.3.1 (Applied Biosystems, CA, United States) with processing on an automated ABI 3130xl sequencer (Applied Biosystems) using Sanger’s method. All sequence data were spliced and cleaned using Sequencher v5.1 (Gene Codes Corporation).

### Genotype identification and analysis

Sequences were edited in AliView software and aligning using the Los Alamos HIV Align tool with HXB2 reference sequences. The phylogenetic tree was approximated using the maximum likelihood method with general time-reversible (GTR) modeling with RAxML (version 8) software [20] and Figtree v1.4.3. Identification of HIV-1 genotypes was perform using the phylogenetic tree and the Stanford REGA HIV-1 Subtyping Tool 3.0 [21]. If the results of the REGA tool were ‘Recombination’, ‘Recombination-like’, ‘potential-Recombination’, ‘check the report’, the phylogenetic tree was used for confirmation.

### Drug resistance mutations and analysis

TDRMs were defined as the proportion of surveillance drug resistance mutations (SDRMs), identified by the Stanford Calibrated Population Resistance (CPR) tool 8.0 (last updated on 2019-07-01), according to the WHO-2009 SDRM list [22]. The Stanford HIVDB Program 8.9.1 (last updated on 2019-10-25, https://hivdb.stanford.edu/hivdb/) was used to score resistance to protease inhibitors (PIs), nucleoside reverse transcriptase inhibitors (NRTIs) and nonnucleoside reverse transcriptase inhibitors (NNRTIs) [23]. Sequences were determined to be susceptible (< 15, including potential resistance) or resistant (15 ≤ low< 30, 30 ≤ medium< 60, or high> 60) based on their scores.

### Statistical analysis

Statistical analyses were perform using R 3.6.2 and R-Studio 1.1.463. The Chi-square test or Fisher’s exact test was used to verify differences in the distribution of demographic and clinical characteristics and genotypes and report proportions of TDRMs or network inclusion by participant characteristics (for example, nationality, educational status, etc.). The Cochran-Armitage trend test was used to test trends such as TDR trend and the proportion of genotypes changed. When appropriate, *p* < 0.05 was defined as statistically significant.

## Results

### Demographic and clinical characteristics of ART-naïve youths at the China-Myanmar border

Overall, the *pol* gene was amplified successfully from 573 untreated youths with HIV-1; of these youths, 351 (61.26%) were male, and 222 (39.74%) were female. The most common infection route was sex among heterosexuals (70.51%), followed by PWID (19.20%) and MSM (8.9%). The proportion of the MSM population is increasing annually (1 to 14%, *p* < 0.001). More than half of the youths were single (58.81%), and the rest were married/cohabiting (37.35%) or divorced (3.84%). The subjects were Chinese (55.67%) or Burmese (44.33%). The proportion of PWID was higher among Burmese than among Chinese subjects (29.13% vs. 11.29%, *p* < 0.001). The educational status of the subjects was mainly primary education (28.27%) and junior high school education (27.23%). The Chinese subjects had a higher rate of junior high school education (34.84%) than the other subjects, and the Burmese subjects had a higher illiteracy rate (38.58%) (Table 1).

**Table 1:** Clinical characterizes of untreated 16 ~ 25y youths infected HIV during 2009 ~ 2017

	2009 ~ 2011 *n* = 115	2012 ~ 2013 *n* = 113	2014 ~ 2015 *n* = 113	2016 ~ 2017 *n* = 232	Total *N* = 573 (100%)
**Gender**					
Male	53	54	71	173	351(61%)
Female	62	59	42	59	222(39%)
**Nationality**					
China	90	64	47	118	319(56%)
Myanmar	25	49	66	114	254(44%)
**Route of transmission**					
Heterosexual	88	80	78	158	404(71%)
IDU	19	26	25	40	110(19%)
MSM	1	7	9	34	51(9%)
Unknown	7	0	1	0	8(1%)
**Marriage status**					
Single	51	53	72	161	337(59%)
Married	56	57	37	64	214(37%)
Divorce\bereavement	8	3	4	7	22(4%)
**Ethnicity**					
Han	42	47	30	41	160(28%)
Minorities	73	66	83	191	413(72%)
**Education**					
Illiteracy	24	29	22	40	115(20%)
Primary school	46	29	28	59	162(28%)
Junior school	38	39	38	41	156(27%)
senior school	4	2	7	20	33(6%)
College and Above	3	4	3	5	15(3%)
Unknown	0	10	15	67	92(16%)
**Job**					
Jobless	11	12	14	21	58(10%)
Farmer	84	76	53	107	320(56%)
Business services	13	4	3	8	28(5%)
Other	7	9	27	25	68(12%)
Unknown	0	12	16	71	99(17%)

### High HIV genotype diversity among ART-naïve youths in China-Myanmar border areas

**Fig. 1:**
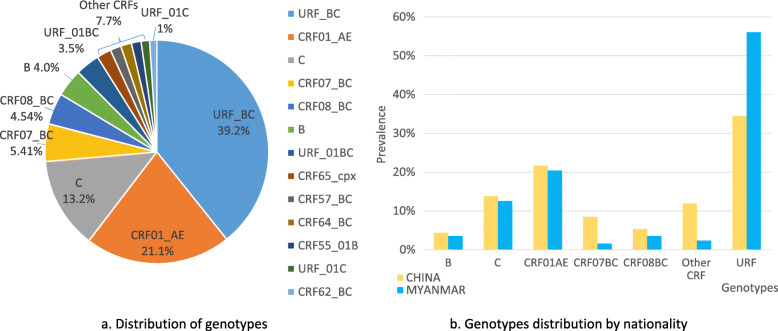
Phylogenetic and genotypic of HIV-1 *pol* genes. **a**. Distribution of genotypes according to HIV-1 *pol* genes. **b**. Genotypes distribution by nationality. URF is higher in Burmese than in Chinese (31.89% vs. 21.63%, *p* < 0.05), and CRF07BC are higher in Chinese than in Burmese (8.46% vs. 1.57%, *p* < 0.01)

The Stanford REGA online tool [21] was used to classify genotypes, and the classification was rechecked in the phylogenetic tree. There was a high distribution of URFs (44.08%), including URF_BC (39.20%), URF_01BC (3.48%), and URF_01C (1.22%), and 38.68% CRF: including CRF01_AE (21.12%), CRF07_BC (5.41%), CRF08_BC (4.54%), CRF5501B (1.39%), CRF57BC (1.57%), CRF62BC (1.05%), CRF65cpx (2.09%) and CRF64BC (1.57%) (Fig. 1a), and 13.24% subtype C and 4.04% subtype B. The genotypes changed over time: subtypes B and C decreased from 2009 ~ 2017, while those of CRF01AE, CRF07BC and URFs continued to increase. According to the route of infection, the proportion of URF in PWID was higher than that observed in people who were infected with HIV-1 through sexual transmission (heterosexuals and MSM), but the proportions of CRF01AE and CRF08BC in cases of sexual transmission were high (*p* < 0.001). There are different HIV infection routes and genotypes between China and Myanmar, such as high URF in Burmese compared to Chinese (34.48% vs. 56.08%, *p* < 0.05) and high CRF07_BC and other CRFs in Chinese compared to Burmese (8.46% vs. 1.57, 11.91% vs. 2.35% *p* < 0.01) (Fig. 1b).

### The HIV TDR among ART-naïve youths at the China-Myanmar border increased significantly from 2009 to 2017

**Fig. 2:**
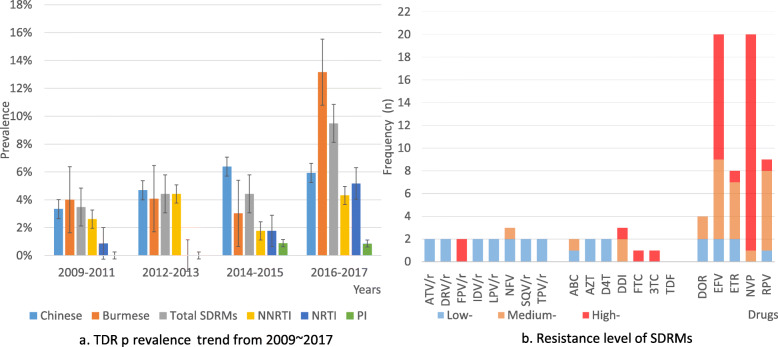
Resistance level and TDR prevalence trend. **a**. TDR prevalence trend from 2009 ~ 2017. Rates (mean, Std. Error, 95% CI) of TDR in Chinese: 2009–2011 (3/90, 3.30%, Std. Error: 1.9, 0.00% ~ 7.60%), 2012–2013 (3/64, 4.70%, Std. Error: 2.66, 0.00% ~ 10.9%), 2014–2015 (3/47, 6.4%, Std. Error: 3.6, 0.00% ~ 14.6%), 2016–2017 (7/118, 5.90%, Std. Error: 2.20, 1.80% ~ 10.7%); Burmese: 2009–2011 (1/25, 4.00%, Std. Error: 4.00, 0.00% ~ 13.6%), 2012–2013 (2/49, 4.10%, Std. Error: 2.8, 0.00% ~ 10.4%), 2014–2015 (2/66, 3.00%, Std. Error: 2.1, 0.00% ~ 7.70%), 2016–2017 (15/114, 13.20%, Std. Error: 3.10, 7.40% ~ 19.7%). **b**. Resistance levels of TDRMs. The degrees of resistance of the observed TDRMs to 20 ART drugs from the Stanford HIVDB program are distinguished by colors. The effects of other non-SDRMs in the sequence are excluded

Thirty-six (36/573, 6.28%) (2009 ~ 2011: 3.48%, 2012 ~ 2013: 4.42%, 2014 ~ 2015: 4.42%, and 2016 ~ 2017: 9.48%) youths with TDRMs among the 573 untreated youths with HIV-1 were identified by the CPR program. TDR increased significantly from 2009 to 2017 (3.48 to 9.48%, *p* < 0.05, Cochran-Armitage trend test). TDR among Chinese increased from 3.33 to 5.93% (*p* = 0.37, Cochran-Armitage trend test), and the highest percentage appeared in 2014 ~ 2015 (6.38%). The TDR among Burmese increased significantly from 4.00 to 13.16% (*p* < 0.05, Cochran-Armitage trend). TDR to NNRTIs (3.9 to 4.31%, *p* = 0.57) and PIs (0 to 0.86%, *p* = 0.20) increased from 2009 to 2017. Moreover, the resistance to NRTIs increased significantly (0.87 to 5.17%, *p =* 0.004, Cochran-Armitage trend test) (Fig. 2a).

Overall, the average TDR to NNRTIs, NRTIs, and PIs was 3.49, 2.62, and 0.52%, respectively. Most (94.4%, *n* = 34) of HIV-1 patients with TDRM displayed a single drug class resistance mutation. Only two (5.6%) strains harbored both NRTI and NNRTI mutations. No HIV-1 strain with TDRMs to triple classes of drugs was found in this study. Y181I/C and K103N were found in 7 and 9 infected individuals, respectively (Table 2). We used the Stanford HIVdb tool to assess the clinical impact of these mutations (excluding other polymorphism sites). Among NNRTIs, resistance to doravirine (DOR), etravirine (ETR), and rilpivirine (RPV) occurred mainly at a moderate level (50, 63, and 78%, respectively), and efavirenz (EFV) and nevirapine (NVP) had high-level resistance (55 and 95%). Among NRTIs, except M184I (1), L74I (1), D67N (1), and T215I (1), the remaining mutations (75%, 12/16) only conferred potential resistance (score < 15) to NRTIs. Only three youths carried TDRMs associated with PI resistance, and the I54M site caused broad resistance to PI drugs (Fig. 2b).

**Table 2:** Prevalence of HIV Transmitted Drug Resistance Mutations among untreated 16 ~ 25y youths in Dehong during 2009 ~ 2017

Type	DRM (%)
**Total TDRMs**	38^a^
**NRTI_TDRMs**	15(2.62%)
D67E	4(0.70%)
D67N	1(0.17%)
F77L	3(0.52%)
K219N	3(0.52%)
L74I	1(0.17%)
M184I	1(0.17%)
T215I	1(0.17%)
T69D	1(0.17%)
**NNRTI_TDRMs**	20(3.49%)
G190A	1(0.17%)
K101E	1(0.17%)
K103N	9(1.57%)
V106M	2(0.35%)
Y181C	7(1.22%)
**PI_TDRMs**	3(0.52%)
I54M	2(0.35%)
M46I	1(0.17%)

^a^ “Heterosexual” and “2016–2017” have two infectors who have two classes of TDRMs

### Correlates of CD4 count and TDRMs among ART-naïve youths in the China-Myanmar border

The CD4 counts of 461 (80.45%) of the youths in the study were recorded. The average CD4 count was 483/mm^3^ 95% CI: 466.31 ~ 513.87/mm^3^. To examine the relationship between CD4 count and TDRMs, we divided subjects into two groups: with or without TDRMs. The mean CD4 count was significantly lower in youths with TDRMs (372.57/mm^3^, 95% CI: 300.44 ~ 444.69/mm^3^) than youths without TDRMs (496.08, 95% CI: 471.91 ~ 520.25/mm^3^). K103N and Y181C were the most common TDRMs in NNRTI. We divided subjects into the K103N group, Y181C group, NRTI group, PI group, and without TDRMs group. The K103N group, Y181C group, and NRTI_TDRMs group had lower mean CD4 counts (337/mm^3^95% CI: 246.42 ~ 427.57/mm^3^, 385/mm^3^ 95% CI: 132.54 ~ 637.85/mm^3^, and 363.6/mm^3^95% CI: 260.51 ~ 489.62/mm^3^, and 434/mm^3^95% CI: 130.69 ~ 737.30/mm^3^, respectively) than the youths without TDRMs group (Table 3). None of the other characteristics or demographic information correlated with TRDMs (supplement Table S2).

**Table 3:** Correlates of CD4 count and DRMs among ART-naïve youths in the China-Myanmar border

	N	Mean (mm^3^)	Standard Error of Mean (mm^3^)	95% CI for Mean		*P*
				Lower Bound (mm^3^)	Upper Bound (mm^3^)	
TDRMs						***0.015***
Without TDRMs	431	496.08	12.13	471.91	520.25	
TDRMs	30	372.57	34.05	300.44	444.69	
NNRTI_TDRMs						*0.226*
Without TDRMs	431	496.08	12.13	471.91	520.25	
With K103N mutation	9	337.00	39.05	246.42	427.57	
With Y181C mutation	5	385.20	91.00	132.54	637.85	
Other mutation	2	514.50	NA	NA	NA	
NRTI_TDRMs						*0.064*
Without TDRMs	431	496.08	12.13	471.91	520.25	
NRTI_TDRMs	13	363.60	54.39	260.51	489.62	
PI_TDRMs						*0.674*
Without TDRMs	431	496.08	12.13	471.91	520.25	
PI_TDRMs	3	434.00	82.70	130.69	737.30	

The CD4 counts of 461 (80%) of the youths in the study were recorded. The results of “Standard Error of Mean” and “95% CI for Mean” are based on 1000 bootstrap samples

## Discussion

The city of Dehong is located in the China-Myanmar border area near the “Golden Triangle” and is a hotspot of HIV transmission and recombination, with a strong impact on the HIV-1 epidemic in China [12, 24]. From 2009 to 2017, 10,832 people were newly reported with HIV-1 infection at the Dehong border of China, 2210 (20.40%) of whom were youths (< 25 y). As youths are more likely to have recent infections and are highly representative of TDR, [16–18] we analyzed the TDR and genotype of untreated youths (16 ~ 25 y) newly diagnosed with HIV-1 infections over a 9-year period in Dehong.

The distribution of HIV-1 genotypes in China primarily includeCRF01AE, CRF07BC, CRF08BC, and B, while C, URF, and CRFs account for only a small proportion [9]. However, the distribution of genotypes differs in Dehong, which has a high prevalence of URFs [15, 25]. Similar to previous studies, the distribution of HIV genotypes in this study was diverse and complex. Notably, the prevalence of genotypes B and C decreased annually, and CRF01AE, CRF07BC, and URFs continues to increase. We also found that the proportion of URFs in Burmese and PWID populations was significantly higher than other populations. This prevalence result may indicate that due to the influence of drug injection in the “Golden Triangle”, the presence of HIV-1 recombination networks occurred early among PWID in Dehong [26–28]. This has had a long-term impact on the HIV-1 epidemic in this area and made Dehong a hotspot for HIV recombination.

Previous studies indicated that frequent recombination was more effective than mutation in spreading drug resistance mutations [29, 30]. Frequent communication around the China-Myanmar border has increased the frequency of recombination [15, 31]. However, this previous study was based on patients after treatment. Recombination allows the genome to combine beneficial mutations that existed before, which is conducive to the survival of viruses in the ARV. Our subjects were treatment-naïve, and there was no choice pressure of ARV drugs. This factor may be why the connection between TDR and reorganization was not significant (*p* = 0.793), despite the increasing reorganization. Overall, the average prevalence of TDR was 6.28%, which exceeds the 5% moderate prevalence level. Since the early years of ART scale-up, TDR strains of HIV are likely to be limited, and all youths were ART-naïve. The total number of TDRMs was small (*n* = 36), which may result in a statistical bias. We increased the sample capacity in 2016–2017 and observed a significant increase in TDR. Notably, the TDR was 9.48% in 2016–2017, which is significantly higher than the average TDR prevalence in China [3] and Myanmar [2]. The TDR in this study does not represent the average resistance level in China and Myanmar but it indicates the TDR increase among youths in hotspots of HIV transmission and recombination. In this study, found no significant difference in TDR prevalence between Burmese and Chinese subjects. The prevalence of TDR in Chinese subjects increased from 2009 to 2017 (from 3.92 to 5.93%), the prevalence of TDR in Burmese migrants increased significantly from 2010 to 2017 (from 4.00 to 13.16%). Burmese migrants are a key population for HIV prevention in this region.

Resistance to NNRTIs (2.92%) was the most frequent TDRM. Among these mutations, K103N (*n* = 9) and Y181C/I (*n* = 7) were the most common TDRMs. These two mutations caused a high level of drug resistance to first-line treatment drugs (EFV and NVP). Among NRTI-related TDRMs (2.34%), most (75%, 12/16) exhibited only potential resistance. Azidothymidine (AZT), lamivudine (3TC), and tenofovir (TDF), as first-line NRTI drugs in China, have meager rates of transmission resistance (0.30, 0.15, and 0%, respectively). However, unlike the findings reported in other areas, [3, 32–37] NRTI resistance showed the most significant increase (from 0 to 5.17%) from 2009 to 2017 in this study. Although the prevalence of TDR to PIs (0.44%) was significantly lower than the prevalence of TDR to NNRTIs/NRTIs, the I54M mutation caused universal resistance to PI drugs. These results suggest the need for considering resistance testing before initiating ART. We did not investigate integrase inhibitor (INSTI) TDR; these sequences were previously amplified and stored by our laboratory, and the primers did not include the INSTI region because INSTI drugs are not widely used in China and Myanmar. We will continue to increase the sample capacity and to monitor TDR (including INSTI) in the China-Myanmar border region.

Previous studies [38, 39] associated TDRMs with high CD4 counts, but our research found that HIV-1-infected youths with TDRMs had low CD4 counts. This discrepancy was also found in at least two other studies [40, 41]. A 2019 study [42] suggested that the detection of primary resistance was not associated with the speed of CD4 decline. We divided these six studies into two groups based on whether CD4 decreased. There were not many similarities in the resistance sites within the group. Notably, all of the studies had limitation. Infected persons at any stage of infection can enter the queue. The decline of CD4 was likely related to different stages of the disease course. In our study, the distribution of transmission routes and genotypes were similar to the overall HIV-1 situation in this region. Demographic information has no correlation with TRDMs, which minimized the error caused by sampling. Moreover, our survey objects were newly reported ART-naïve youths, which reduces the error of infection time as possible and excludes individuals with long-standing infections or prior ART. Therefore, this relationship between CD4 count and TDRMs may be generalized to individuals infected with HIV-1 at the China-Myanmar border. We compared the CD4 count between NNRTI/NRTI/PI mutation and non-TDRM youths. Although all groups with TDRMs showed a lower mean CD4 count than the group without TDRMs, the difference was not statistically significant. Due to the small number of other TDRMs, we could not determine the correlation between other single TDRMs and CD4 counts. In summary, large sample size and more epidemiological data are required to evaluate the potential role of three classes of TDR (NNRTI/NRTI/PI) or K103N, Y181C in affecting the decline of CD4 count.

## Conclusion

TDR increased two-fold and exceeded the 5% moderate prevalence level from 2009 to 2017 among untreated youths in the China-Myanmar border, primarily resistance to NNRTIs and NRTIs. HIV-1 genotypes are diverse and complex, with URFs, CRF01AE as the predominant genotypes. The HIV-1 TDR increase and a low CD4 count of patients with TDRMs suggest the need for considering drug resistance before initiating ART in HIV recombination hotspots.

## Supplementary Information

**Additional file 1: Table S1.** Age distribution of newly reported HIV infections in Dehong city, during 2009 ~ 2017. **Table S2.** Demography and clinical characteristics of untreated 16 ~ 25y youths infected HIV with or without TDRMs.

## Data Availability

The datasets used during the current study are available from the corresponding author on reasonable request.

## Abbreviations

HIV: Human immunodeficiency virus
TDR: Transmitted drug resistance
ADR: Acquired drug resistance
PLWH: People living with HIV
WHO: World Health Organization
ART: Antiretroviral therapy
NNRTI: Non-nucleoside reverse-transcriptase inhibitor
NRTI: Nucleoside reverse-transcriptase inhibitor
PI: Protease inhibitor
PWID: People who injected drug
PrEP: Pre-exposure prophylaxis
EFV: Efavirenz
NVP: Nevirapine
FTC: Emtricitabine
3TC: Lamivudine
ABC: Abacavir
DDI: Didanosine
D4T: Stavudine
TDF: Tenofovir
AZT: Azidothymidine
DRV/r: Darunavir
LPV/r: Lopinavir
ATV/r: Atazanavir
DRM: Drug resistance mutation
HIVDR: HIV Drug Resistance Database
95% CI: 95% Confidence interval
