# Supplementary material for: Increase in HIV-1-transmitted drug resistance among ART-naïve youths at the China-Myanmar border during 2009 ~ 2017
Source: BMC Infect Dis. 2021 Jan 21;21:93. doi: 10.1186/s12879-021-05794-5 (PMC7818912; doi:10.1186/s12879-021-05794-5)
Supplement: Supplementary file 1 — Additional file 1: Table S1. Age distribution of newly reported HIV infections in Dehong city, during 2009 ~ 2017. Table S2. Demography and clinical characteristics of untreated 16 ~ 25y youths infected HIV with or without TDRMs. [file 12879_2021_5794_MOESM1_ESM.docx]

Table S1 Age distribution of newly reported HIV infections in Dehong city, during 2009~2017

|  | 2009~2011 | 2012~2013 | 2014~2015 | 2016~2017 | Total |
| --- | --- | --- | --- | --- | --- |
| Total of newly reported individuals(n) | 4120 | 2480 | 2143 | 2089 | 10832 |
| Total of <25y youths(n) | 847 | 499 | 420 | 444 | 2210 |
| Samples which according to the sampling standard (n) | 140 | 138 | 148 | 240 | 666 |
| *Pol* genes were amplified and TDR analysis was performed successfully | 115 | 113 | 113 | 232 | 573 |

Table S2 Demography and clinical characteristics of untreated 16~25y youths infected HIV

with or without TDRMs

|  | Total | Without TDR | TDR | Std. Error of TDR | 95% CI of TDR | *X^2^* | *p* |
| --- | --- | --- | --- | --- | --- | --- | --- |
| **Sex (n)** |  |  |  |  |  | *3.057* | *0.08* |
| Male | 351 | 324(92%) | 27(8%) | 1% | 5~11% |  |  |
| Female | 222 | 213(96%) | 9(4%) | 1% | 2~7% |  |  |
| **Marriage status (n)** |  |  |  |  |  | *1.793* | *0.408* |
| single | 337 | 312(93%) | 25(7%) | 1% | 5~10% |  |  |
| Marriage | 214 | 204(95%) | 10(5%) | 1% | 2~8% |  |  |
| Divorce | 22 | 21(95%) | 1(5%) | 5% | 0~15% |  |  |
| **Route (n)** |  |  |  |  |  | *2.701* | *0.440* |
| Heterosexual | 404 | 382(95%) | 22(5%) | 1% | 3~8% |  |  |
| IDU | 110 | 100(91%) | 10(9%) | 3% | 4~15% |  |  |
| MSM | 51 | 47(92%) | 4(8%) | 4% | 2~16% |  |  |
| Unknown | 8 | 8(100%) | 0(0%) | / | / |  |  |
| **HIV-1 genotype (n)** |  |  |  |  |  | *3.128* | *0.793* |
| CRF01AE | 121 | 110(92%) | 11(8%) | 3% | 3%~13% |  |  |
| CRF07BC | 31 | 29(94%) | 2(6%) | 4% | 0~15% |  |  |
| CRF08BC | 26 | 25(96%) | 1(4%) | 4% | 0~13% |  |  |
| B | 23 | 22(96%) | 1(4%) | 4% | 0~14% |  |  |
| C | 76 | 75(99%) | 1(1%) | 1% | 0~4% |  |  |
| URF | 252 | 235(93%) | 17(7%) | 2% | 4~10% |  |  |
| Other | 44 | 41(93%) | 3(7%) | 4% | 0~15% |  |  |
| **Nationality (n)** |  |  |  |  |  | *1.962* | *0.161* |
| Chinese | 319 | 303(95%) | 16(5%) | 1% | 3~8% |  |  |
| Myanmar | 254 | 234(92%) | 20(8%) | 2% | 5~11% |  |  |
